# Effect of Isoquercitrin on Free Fatty Acid-Induced Lipid Accumulation in HepG2 Cells

**DOI:** 10.3390/molecules28031476

**Published:** 2023-02-03

**Authors:** Sou Hyun Kim, Chawon Yun, Doyoung Kwon, Yun-Hee Lee, Jae-Hwan Kwak, Young-Suk Jung

**Affiliations:** 1 Department of Pharmacy, College of Pharmacy, Research Institute for Drug Development, Pusan National University, Busan 46241, Republic of Korea; 2 College of Pharmacy, Jeju Research Institute of Pharmaceutical Sciences, Jeju National University, Jeju 63243, Republic of Korea; 3 College of Pharmacy, Research Institute of Pharmaceutical Sciences, Seoul National University, Seoul 08826, Republic of Korea; 4 College of Pharmacy, Chungbuk National University, Cheongju 28160, Republic of Korea

**Keywords:** nonalcoholic fatty liver disease, isoquercitrin, lipid metabolism, AMP-activated protein kinase, sterol regulatory element-binding transcription factor 1, fatty acid synthase, endoplasmic reticulum stress

## Abstract

Liver metabolic disorders and oxidative stress are crucial factors in the development of nonalcoholic fatty liver disease (NAFLD); however, treatment strategies to combat NAFLD remain poorly established, presenting an important challenge that needs to be addressed. Herein, we aimed to examine the effect of isoquercitrin on lipid accumulation induced by exogenous free fatty acids (FFA) using HepG2 cells and elucidate the underlying molecular mechanism. The cells were exposed to 0.5 mM FFA to induce intracellular lipid accumulation, followed by co-treatment with isoquercitrin to confirm the potential inhibitory effect on FFA-induced lipid production. HepG2 cells exposed to FFA alone exhibited intracellular lipid accumulation, compromised endoplasmic reticulum (ER) stress, and enhanced expression of proteins and genes involved in lipid synthesis; however, co-treatment with isoquercitrin decreased the expression of these molecules in a dose-dependent manner. Furthermore, isoquercitrin could activate AMP-activated protein kinase (AMPK), a key regulatory protein of hepatic fatty acid oxidation, suppressing new lipid production by phosphorylating acetyl-CoA carboxylase (ACC) and inhibiting sterol regulatory element-binding transcription factor 1 (SREBP-1)/fatty acid synthase (FAS) signals. Overall, these findings suggest that isoquercitrin can be employed as a therapeutic agent to improve NAFLD via the regulation of lipid metabolism by targeting the AMPK/ACC and SREBP1/FAS pathways.

## 1. Introduction

Nonalcoholic fatty liver disease (NAFLD) is the most common chronic liver disease worldwide. Clinically, NAFLD has been associated with a wide spectrum of liver abnormalities, ranging from steatosis to nonalcoholic steatohepatitis (NASH), fibrosis, and cirrhosis [1]. NAFLD is characterized by the excessive accumulation of fat in the liver owing to factors other than alcohol abuse [2,3]. NAFLD is closely associated with obesity, diabetes, hyperlipidemia, and insulin resistance [4]. In the liver, accumulated free fatty acid (FFA) functions as a raw material for neutral fat synthesis. In addition, excessive carbohydrate intake rapidly increases FFA production in the body, which moderately enhances FFA absorption in the liver and triglyceride biosynthesis from carbohydrate metabolism. As a result, the amount of neutral fat in the liver rapidly decreases during decomposition when compared with the amount synthesized, resulting in NAFLD. 

Polyphenols comprise a variety of plant-derived compounds that share the chemical properties of water solubility and are widely found in red fruits, tea, coffee, red wine, and dark chocolate. Polyphenols can prevent oxidative stress, promote β-oxidation of fatty acids, and control insulin resistance [5,6]. In addition, polyphenol compounds have been shown to inhibit lipogenic enzyme activity and control new lipid synthesis [7,8]. Given that inflammation and oxidative stress are primary risk factors involved in the development of NAFLD [9], recent studies have reported that consuming a polyphenol-rich diet could help prevent and treat NAFLD [10]. Accordingly, considerable efforts have been made to assess the effectiveness of polyphenols on insulin resistance and metabolic pathologies in NAFLD. Although several drugs were found to improve NAFLD in animals and humans, side effects, such as abdominal pain, constipation, nausea, fatigue, diarrhea, headache and pruritus, have also been reported [11]. 

**Figure 1:**
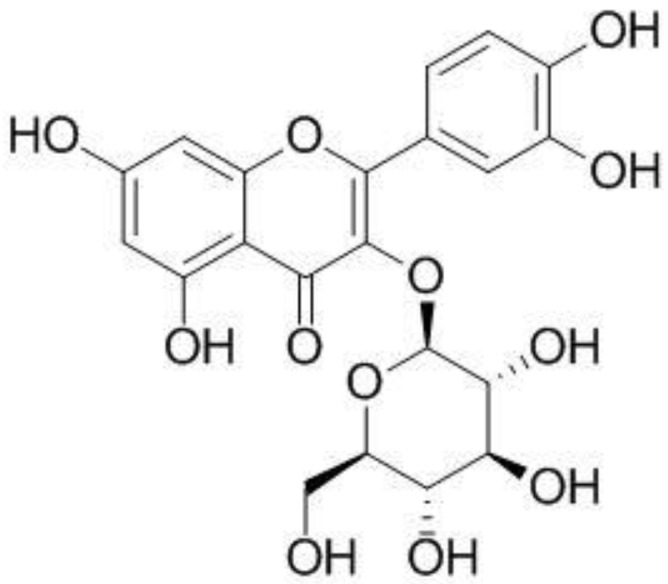
Chemical structure of isoquercitrin.

Isoquercitrin (synonym: isoquertcitin) is a polyphenol with a glycosidic bond in the third position of quercitrin (Figure 1). Isoquercitrin has been isolated from various plant species, including *Mangifera indica* (mango) and *Rheum rhabarbarum* (noble rhubarb). In addition, isoquercitrin is known to be present in the leaves of *Vestia foetida*, *Annona squamosa*, and *Camelia sinensis* [12]. Although poor water solubility hinders absorption from food and following oral intake [13], isoquercitrin is known for a variety of physiological activities. For example, isoquercitrin reportedly exhibits a wide range of biological functions, such as antioxidant and antiviral effects, blood pressure drops, and anti-inflammatory effects, both in vivo and in vitro [14], including reduced lipid peroxidation and reactive oxygen species (ROS) levels [15,16]. It was reported that pretreatment with isoquercitrin in the mice significantly weakened not only liver oxidative and nitrosative stress but also centrilobular necrosis induced by acetaminophen (APAP), suggesting the strong antioxidant activity of isoquercitrin in the liver [17]. Moreover, this polyphenol could inhibit the production of inflammatory cytokines, TNF-α, iNOS, IL-6, and IL-1 by blocking the NF-κB and MAPK pathways [17]. Therefore, it was suggested that the anti-oxidative and anti-inflammatory activities of isoquercitrin could be the mechanism of liver protection against APAP.

The endoplasmic reticulum (ER), an abundant intracellular organelle in hepatocytes, is responsible for protein synthesis and folding. ER also plays an important role in maintaining calcium homeostasis and biosynthesis of sterols, carbohydrates, and lipids [18,19,20]. It has been reported that abnormalities in ER functions induced by cellular stresses, such as oxidative stress, can be closely associated with the dysregulation of lipid metabolism in NAFLD [21,22,23]. ER stress is the status of unfolded/misfolded protein accumulation in the ER, and severe, and prolonged ER stress can promote apoptotic cell death [21,22,23]. Hepatic ER stress can be induced by excessive lipid exposure, and thus this cellular stress plays an important role in the pathogenesis of NAFLD [21,22,23]. Meanwhile, sterol regulatory element binding proteins (SREBPs) are transcription factors that mediate fatty acid synthesis, cholesterol homeostasis and lipid production [24,25]. Stearoyl-CoA desaturase-1 (SCD) is a protein that produces unsaturated fatty acids, which can be used for triglyceride (TG) synthesis [26,27]. Adenosine 5′-monophosphate (AMP)-activated protein kinase (AMPK) is a key protein in the regulation of hepatic lipid metabolism by inhibiting the SREBP-1c-mediated lipid synthesis and activating fatty acid oxidation [28,29,30]. Inhibition of AMPK increases the expression of SREBP-1c, which in turn induces lipogenic proteins such as ACC, FAS, and SCD. Activated AMPK phosphorylates ACC to inhibit the synthesis of malonyl-CoA that inhibits mitochondrial carnitine palmitoyltransferase-1 (CPT-1). Because CPT-1 facilitates fatty acid uptake into mitochondria, AMPK activation can enhance mitochondrial fatty acid oxidation [28,29,30]. Therefore, ER-stress and AMPK-mediated signaling pathways can be the therapeutic targets of NAFLD [31,32,33].

Isoquercitrin has shown hepatoprotective effects in NAFLD via its anti-oxidative and anti-inflammatory actions. Although the anti-steatogenic effect of isoquercitrin was previously reported, the exact mechanism is not fully understood. Therefore, the aim of the present study was to identify the lipid-lowering mechanism of isoquercitrin in an in vitro NAFLD model using human liver HepG2 cells treated with FFA. The results show that isoquercitrin can significantly inhibit FFA-induced hepatic lipid accumulation via the regulation of lipid metabolism, ER stress, and MAPK signaling pathway.

## 2. Results

### 2.1. Isoquercitrin Suppress FFA-Induced Lipid Accumulation in HepG2 Cells

**Figure 2:**
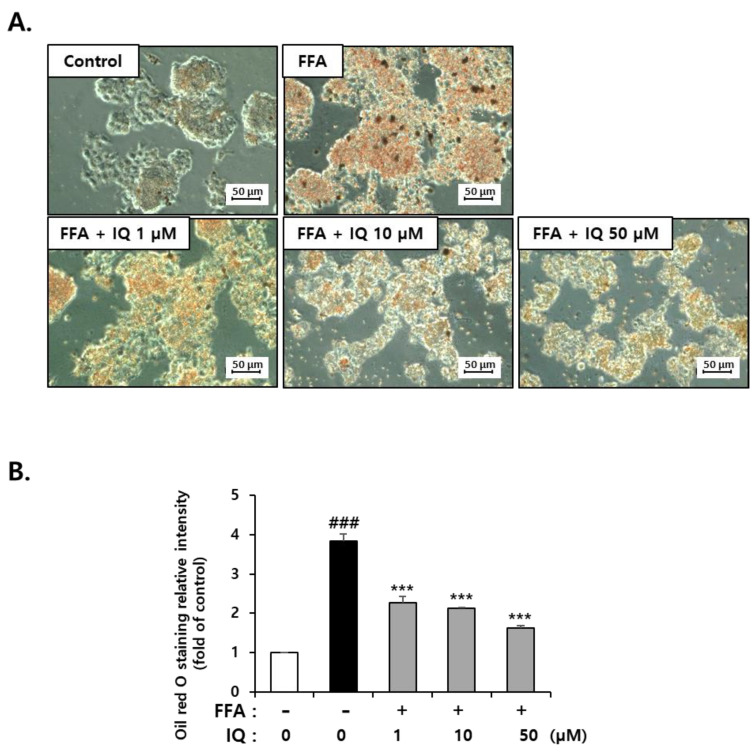
Isoquercitrin inhibits FFA-induced lipid accumulation in HepG2 cells. (**A**) Oil-Red-O staining after FFA and isoquercitrin treatment. (**B**) Oil-Red-O staining was quantitated by total cell number, and the data are presented from three independent experiments. Each value represents the mean ± SD. ^###^ *p* < 0.001, significant versus control; *** *p* < 0.001, significant versus FFA alone. FFA, free fatty acid.

HepG2 cells are widely used in in vitro NAFLD models because they have the characteristics of hepatocytes that accumulates intracellular lipids by FFA treatments such as oleic acid and/or palmitic acid [9,34,35]. Many studies have reported that the exposure of HepG2 cells to 0.5–1 mM of FFA for 24 h results in significant lipid accumulation [16,36,37]. To evaluate the involvement of isoquercitrin in regulating lipid metabolism, HepG2 cells were pretreated with isoquercitrin for 6 h with doses from 1 to 50 μM and co-treated with FFA (0.5 mM) composed of oleic acid and palmitic acid (2:1) for 24 h. As shown in Figure 2A, treatment with increasing doses of isoquercitrin consistently decreased Oil-Red-O staining when compared with that in FFA-only treated samples. Oil-Red-O quantitation by eluate absorbance normalized to total cell number revealed a dose-dependent decrease with isoquercitrin co-treatment (Figure 2B). These results suggested that isoquercitrin could suppress FFA-induced lipid accumulation in HepG2 cells. 

### 2.2. Isoquercitrin Suppresses FFA-Induced Endoplasmic Reticulum (ER)-Stress in HepG2 Cells

**Figure 3:**
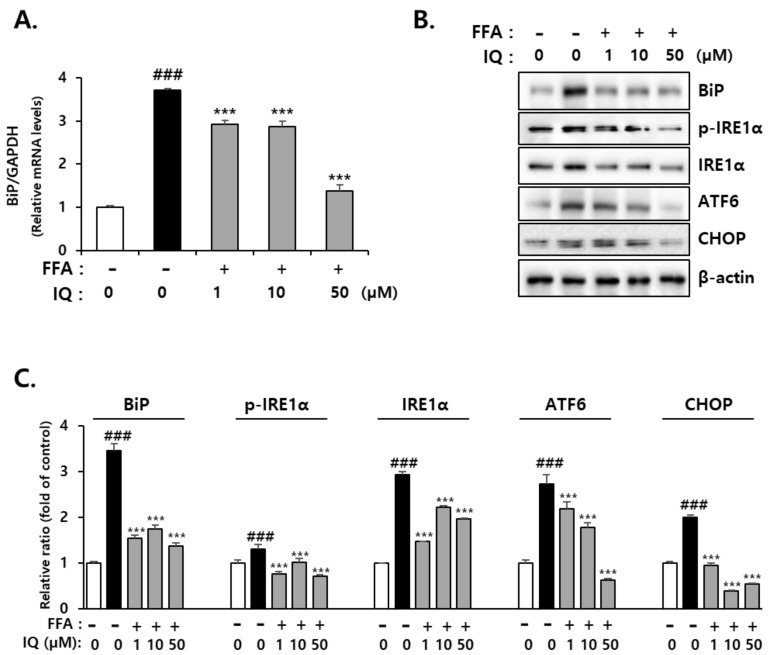
Isoquercitrin inhibits the expression of ER-stress-associated markers. (**A**) mRNA expression of BiP. The relative ratio of each protein was normalized by GAPDH gene expression. (**B**) Expression of ER stress-associated proteins such as BiP, p-IRE1α, IRE1α, ATF6, and CHOP. (**C**) The relative ratio of each protein was normalized by β-actin. ^###^ *p* < 0.001, significant versus control; *** *p* < 0.001, significant versus FFA alone. ATF6, activating transcription factor 6; BiP, binding immunoglobulin protein; CHOP, C/EBP homologous protein; ER, endoplasmic stress; FFA, free fatty acid; IQ, isoquercitrin; IRE1α, inositol-requiring enzyme 1α.

To clarify the effect of isoquercitrin on FFA-induced cytotoxicity, we examined its role in ER stress. The induction of glucose-regulated protein (GRP78), also referred to as binding immunoglobulin protein (BiP), is an indicator of ER stress [38]. BiP is a central regulator of ER stress, given its role as a major ER guardian with anti-apoptotic properties. BiP can control the activation of intermembrane ER stress sensors, such as inositol-requiring enzyme 1α (IRE1α), protein kinase R (PKR)-like endoplasmic reticulum kinase (PERK), and activating transcription factor 6 (ATF6) through a binding-release mechanism [39]. Isoquercitrin treatment decreased BiP expression in FFA-exposed cells. In addition, FFA treatment enhanced the expression of C/EBP homologous protein (CHOP), a key initiating factor of ER stress-related cell death, which was reduced by isoquercitrin (Figure 3A). Treatment with FFA increased the expression of ATF6, a transcription factor activated by ER stress, along with protein expression of IRE1α and p-IRE1α; these expression levels significantly decreased following co-treatment with isoquercitrin. In addition, the expression levels of representative ER stress marker genes, including BiP and CHOP, were enhanced by FFA, and co-treatment with isoquercitrin significantly reduced these expression levels (Figure 3B).

### 2.3. Isoquercitrin Suppress FFA-Induced Lipid Synthesis-Associated Protein Expression in HepG2 Cells

**Figure 4:**
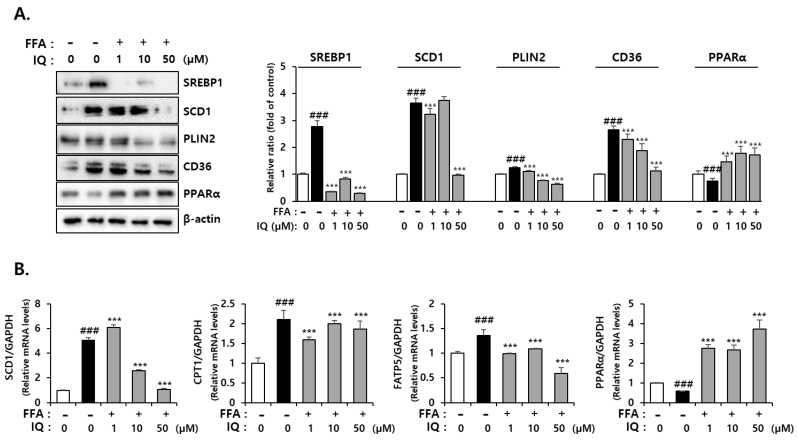
Isoquercitrin inhibits the expression of lipid synthesis-associated proteins and genes. (**A**) Expression of lipid synthesis-associated protein. The relative ratio of each protein was normalized by β-actin expression. (**B**) Expression of lipid synthesis-associated genes. ^###^ *p* < 0.001, significant versus control; *** *p* < 0.001, significant versus FFA alone. CD36, cluster of differentiation 36; FFA, free fatty acid; IQ, isoquercitrin; PLIN2, perilipin 2; PPARα, peroxisome proliferator-activated receptor alpha; SCD1, stearoyl-CoA desaturase 1; SREBP-1, sterol regulatory element-binding transcription factor 1.

The major aspects of NAFLD development include liver inflammation, increased oxidative stress, and fat accumulation [40]. To determine the effect of isoquercitrin on lipid accumulation in FFA-induced NAFLD, we examined protein expression related to fatty acid absorption and lipid export. As shown in Figure 4A, treatment with FFA increased the expression of SREBP-1, known to promote lipid synthesis, and SCD1, known to facilitate lipid accumulation by converting saturated fatty acids to monounsaturated fatty acids [41]. In addition, treatment with FFA increased the expression of cluster of differentiation 36 (CD36), a fatty acid absorption carrier [42], and perilipin2 (PLIN2), which increases lipid droplets [43]. However, co-treatment with isoquercitrin could significantly decrease these protein expression levels. Expression of Peroxisome Proliferator-Activated Receptor alpha (PPARα), which controls the oxidation of fatty acids by promoting peroxisomal and mitochondrial β-oxidation and de novo synthesis of fatty acids [44], was reduced following FFA treatment and enhanced by isoquercitrin co-treatment in a dose-dependent manner. The expression of lipid synthesis-associated genes was also downregulated by isoquercitrin co-treatment in FFA-only treated cells (Figure 4B). Considering PPARα gene expression, similar to protein expression, mRNA expression was decreased in FFA-treated cells but increased in isoquercitrin-treated cells. Our results indicated that isoquercitrin could inhibit FFA-induced lipid accumulation. 

### 2.4. Effect of Isoquercitrin on Hepatic AMPK Signaling

**Figure 5:**
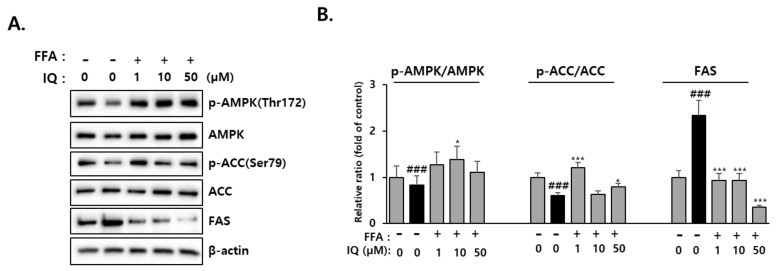
Isoquercitrin enhances AMPK and inhibits ACC/FAS pathway. (**A**) Protein expression of p-AMPK, AMPK, p-ACC, ACC, FAS. (**B**) The relative ratio of each protein was normalized by GAPDH gene expression. ACC, acetyl-CoA carboxylase; AMPK, AMP-activated protein kinase; FAS, fatty acid synthase; FFA, free fatty acid; IQ, isoquercitrin. ^###^ *p* < 0.001, significant versus control; *, *** *p* < 0.05 and 0.001, respectively, significant versus FFA alone.

It is postulated that AMPK can promote the inhibition of lipogenic gene expression in the liver by directly phosphorylating two major transcription factors, carbohydrate-responsive element-binding protein (ChREBP) and SREBP-1 [28]. AMPK inhibits fat production by regulating the phosphorylation of ACC1 and ACC2, which are key regulators of malonyl-CoA synthesis [45]. To evaluate the function of the proteins involved in the AMPK pathway, we examined the protein levels of p-AMPK, AMPK, p-ACC, and ACC to clarify the mechanism through which isoquercitrin inhibits lipid accumulation in HepG2 cells. As shown in Figure 5, compared with FFA-treated cells, isoquercitrin-treated cells displayed significantly increased not only AMPK but also its phosphorylated form (p-AMPK). In addition, treatment with isoquercitrin enhanced p-ACC and p-ACC/ACC ratio, suggesting the inhibition of ACC activity by isoquercitrin-mediated activation of AMPK. Moreover, the FAS protein that was increased by FFA was markedly decreased by isoquercitrin, which could be related to the decreased SREBP-1c [46]. Overall, these results suggested that isoquercitrin downregulated de novo lipogenesis by inhibiting SREBP-1 and activating AMPK pathways resulting in the consequent decrease of ACC and FAS activities.

### 2.5. Activation of Downstream Signaling Molecules of 4-HNE by FFA and Its Rescue Effect of Isoquercitrin

**Figure 6:**
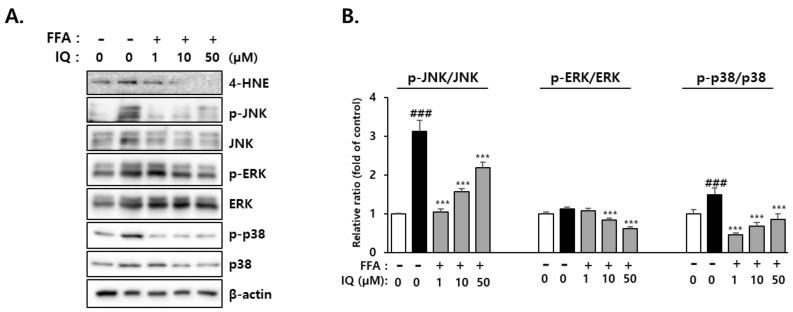
Isoquercitrin inhibits oxidative stress-mediated signaling. (**A**) Protein expression of 4-HNE and oxidative stress-associated signaling molecules. β-actin was used as the loading control. (**B**) The relative ratio of p-JNK/JNK, p-ERK/ERK and p-p38/p38. Each value represents the mean ± SD. ^###^ *p* < 0.001, significant versus control; *** *p* < 0.001, significant versus FFA alone.

4-Hydroxynonenal (4-HNE), a by-product of lipid peroxidation, is known to activate mitogen-activated protein kinases (MAPKs), such as extracellular signal-regulated kinase ERK (ERK1/2), c-Jun N-terminal kinase (JNK), and p38. Activation of MAPKs, including p38 and JNK, reportedly plays a role in FFA-induced lipotoxicity [47]. Our study examined the activation of p38, JNK, and ERK signaling pathways along with the hepatic level of 4-HNE to establish the role of isoquercitrin in FFA-induced intracellular lipid accumulation and its inhibitory effects. Considering ERK, despite the involvement of ERK1/2 in liver metabolism and increased expression during obesity [48], several reports have revealed that ERK1/2 activity remains unaltered [49,50]. In the current study, treatment with FFA elevated the protein levels of p-JNK, p-ERK, and p-p38, which were reduced following co-treatment with isoquercitrin (Figure 6A,B). 

## 3. Discussion

Despite considerable research endeavors, an ideal treatment strategy for combating NAFLD is yet to be established and remains an important unmet challenge. Given the nature of NAFLD, which progresses without substantial symptoms, a lack of early patient management can lead to hepatitis, cirrhosis, and hepatocellular carcinoma [51,52]. NAFLD is a complex disease associated with inflammation, oxidative stress, lipotoxicity, and insulin resistance [19]. Therefore, developing a drug capable of controlling fatty liver and applicable to all patients can pose a substantial challenge, given that each patient exhibits distinct physical characteristics. Accumulated evidence suggests that isoquercitrin-rich foods could benefit patients with NAFLD.

The ER is an intracellular structure that extends from the nuclear membrane and has attached ribosomes [53]. Approximately one-third of proteins are translated from mRNA to protein in the ER, and the post-translational modification process generates an active protein structure [54]. The ER is also involved in lipid and sterol synthesis and plays a critical role in controlling cellular calcium concentrations as a calcium reservoir [55]. However, when immature proteins are introduced beyond the capacity of the ER to modulate the altered cellular environment, ER function is impaired, termed ER stress [56]. FFA treatment increases cell apoptosis by inducing ER stress in certain cells, such as β-TC3, meniscus cells, INS-1, 3T3-L1, and HepG2 cells [57,58,59,60]. Considering these theories, the expression of representative ER stress markers was evaluated to determine the effects of isoquercitrin on the FFA-induced ER stress response. We confirmed that isoquercitrin could inhibit FFA-induced lipid accumulation in HepG2 cells in a dose-dependent manner (Figure 2). In addition, isoquercitrin could protect cells from fatty acid-induced ER stress (Figure 3). Our findings are corroborated by those of several previous studies showing that isoquercitrin can effectively protect the liver from NAFLD-induced damage [17,61]. 

The beneficial effects of isoquercitrin in NAFLD treatment and management via the AMPK pathway have been previously reported [62,63]. In 2020, Kim et al. demonstrated that AMPK could regulate the activity of enzymes involved in fat production, such as ACC, SREBP-1, and FAS, through phosphorylation and dephosphorylation in healthy hepatocytes. However, enhanced AMPK dephosphorylation in NAFLD can cause fatty acid synthesis, thereby inducing fat production and elevated ACC expression while upregulating SREBP-1 via dephosphorylation [64]. SREBP-1 is one of the main factors involved in lipid synthesis and regulates the expression of genes such as FAS and SCD1. Therefore, the isoquercitrin-mediated decreased SREBP-1 expression, as observed in our results (Figure 4), can be considered a reduction in SCD1 expression, accompanied by reduced FAS expression, consistent with previous reports [65,66]. It was reported that isoquercitrin-promoted AMPK activation could enhance AdipoR1 expression and inhibit SREBP-1/FAS expression, resulting in the decrease of lipid accumulation in rat hepatoma H4IIE cells [63]. The results of previous and our studies consistently show that regulation of AMPK/ACC signaling by isoquercitrin can be an important mechanism of its anti-steatogenic effect in the liver cells. Furthermore, the present study reveals that isoquercitrin can also regulate protein and/or mRNA expressions involved in fatty acid metabolism, such as SCD1, CD36, PLIN2, PPARα, CPT1, and FATP5. Our findings, the inhibition of ER stress by isoquercitrin, also can support the results of previous studies demonstrating that isoquercitrin can protect liver cells against lipotoxicity in NAFLD [61,67].

AMPK participates in energy sensing and regulation of homeostasis in vivo [68], playing an important role in the metabolism of glucose uptake and lipid regulation [69]. AMPK, an enzyme that acts as a sensor for maintaining energy homeostasis in cells, is activated when cellular energy decreases during metabolic stress or exercise [70]. Accordingly, ATP depletion and an increased AMP/ATP ratio suppress fatty acid synthesis and cholesterol synthesis, both processes involved in ATP production. As AMPK is activated in the liver, fatty acid and cholesterol synthesis are suppressed, whereas fatty acid oxidation is promoted. Thus, AMPK plays an important role in regulating liver fat metabolism by mediating the synthesis and decomposition of fatty acids. Activation of AMPK inactivates ACC via phosphorylation of ACC, an essential enzyme for fatty acid and cholesterol synthesis, a process known to consume ATP [71]. The ACC enzyme, which synthesizes malonyl-CoA, is an important precursor for fatty acid synthesis and acts as a potential inhibitor of fatty acid oxidation in mitochondria [72]. Accordingly, AMPK-mediated ACC inactivation upon isoquercitrin treatment, as observed in the present study, can reduce the concentration of malonyl-CoA and enhance the activity of carnitine palmitoyltransferase-1, thereby increasing fatty acid oxidation.

Recent reports suggest that mitochondrial dysfunction plays an important role in NAFLD. Enhanced mitochondrial fatty acid oxidation due to excessive fat inflow induces substantial stress on mitochondria, which abnormally increases the transfer of the reducing equivalent from the activated tricarboxylic acid (TCA) cycle to the electron transport system. This increased stress eventually enhances the production of peroxidases, well-known precursors of most ROS, in the mitochondrial respiratory complex [73]. The oxidation of biomolecules, accompanied by this increase in ROS levels, can induce mitochondrial damage by disrupting the electron transport system, eventually causing structural alterations in mitochondria. Hepatocytes suppress the onset of a vicious cycle of mitochondrial dysfunction via the antioxidant system [74]. However, during NAFLD, increasing ROS production results in the excessive expression of catalase, glutathione, and superoxide dismutase, accompanied by enhanced oxidative damage in liver cells when compared with that in normal liver cells [75].

ROS and lipid peroxide products, such as 4-HNE, directly impair mitochondrial DNA and activate mitochondrial apoptosis pathways [76]. NASH development has been associated with a considerable increase in mitochondrial HNE protein expression [77]. Herein, our findings revealed that 4-HNE was markedly increased following FFA exposure, and isoquercitrin reversed this effect (Figure 6). In addition, expression levels of p-p38 and p-JNK were increased in FFA-exposed cells, which were restored following co-treatment with isoquercitrin in a dose-dependent manner. Therefore, isoquercitrin could effectively protect cells from increased ROS levels induced by FFA exposure by suppressing accumulated oxidative stress in cells. Previously, Hassan et al. reported that isoquercitrin inhibits intracellular lipid accumulation by regulating oxidative stress in rat primary hepatocytes and BRL-3A cells [16]. In this study, isoquercitrin reduced FFA-induced oxidative stress in the liver cells but enhanced antioxidant capacity via increasing superoxide dismutase activity [16]. Intriguingly, the addition of hydrogen peroxide to FFA aggravated lipid accumulation in the cells, but the intracellular lipids were significantly reduced by isoquercitrin [16]. Thus, these results indicate that the antioxidant effect of isoquercitrin can be one of the reasons for its lipid-lowering activity. This could also be supported by our results that this polyphenol inhibited FFA-induced 4-HNE formation. Inhibition of oxidative stress is also a well-known mechanism underlying liver protection afforded by various plant-derived polyphenols. In addition, these compounds can downregulate inflammation, prevent lipotoxicity, and improve insulin resistance. Foods rich in isoquercitrin were found to significantly increase liver catalase, glutathione, and superoxide dismutase activities and decrease lipid peroxidation in mice fed a high-fat diet [78,79]. Isoquercitrin primarily protects liver cells by alleviating hepatosteatosis and fibrosis [80,81].

In conclusion, the results of the present study clearly show that isoquercitrin inhibits lipid accumulation induced by FFA in HepG2 cells via proper regulation of lipid metabolism. Inhibition of de novo lipogenesis by SREPB1 and activation of AMPK-mediated signaling pathway can be the mechanisms of the anti-steatogenic effect of isoquercitrin. Specifically, we, for the first time, report that isoquercitrin can ameliorate FFA-induced ER stress and oxidative stress-associated MAPK activation, which could damage the liver cells. Therefore, isoquercitrin can be suggested to be a promising drug candidate for NAFLD.

## 4. Materials and Methods

### 4.1. Cell Culture

Human HepG2 cells were purchased from American Type Culture Collection (Manassas, VA, USA). Cells were grown in Dulbecco’s modified Eagle’s medium (Gibco, Invitrogen, Carlsbad, CA, USA) containing 10% heat-inactivated fetal bovine serum (Hyclone, Logan, UT, USA) and 1% penicillin/streptomycin solution (Hyclone) at 37 °C in a humidified atmosphere of 5% CO_2_. The medium was replaced every 2–3 days for all cell culture assays. Isoquercitrin (quercetin-3-O-β-d-glucopyranoside, ≥98% purity, #17793, Sigma-Aldrich, Inc., St. Louis, MO, USA) was treated to HepG2 cells for 6 h prior to FFA exposure, at the concentrations ranging from 1 to 50 μM which previously showed its lipid-lowering effect in rat primary hepatocytes by 24 h incubation without negative effects [16]. Cells were incubated with 0.5 mM FFA composed of oleic acid (#O7501, Sigma-Aldrich, Inc.) and palmitic acid (#P0007, Tokyo Chemical Industry Co. Ltd., Tokyo, Japan; 2:1) with or without isoquercitrin (1–50 μM) co-treatment for 24 h, and samples were harvested for analysis.

### 4.2. RNA Isolation and Gene Expression Analysis

The primer sets used in the present study are listed in Table 1. To analyze mRNA expression levels, quantitative PCR (qPCR) was performed using a Bio-Rad CFX Connect™ Real-Time PCR Detection System (Bio-Rad Laboratories, Inc., Hercules, CA, USA). TRIzol™ was purchased from Ambion (Life Technologies, Waltham, MT, USA), and Direct-zol™ RNA MiniPrep was purchased from Zymo Research (Irvine, CA, USA). Reverse transcription-qPCR was performed using the SensiFAST SYBR No-ROX Kit (Bioline, London, UK). qPCR was performed using the following program: denaturation at 95 °C for 5 min, followed by 40 repeated cycles of 95 °C, 1 s/60 °C, 30 s/72 °C, 20 s: 79 cycles at 55 °C for 30 s each to generate melting curves. Relative gene expression was analyzed using the Livak method [82]. A normalized expression ratio (2^−ΔΔ*C*t^) was calculated for each target gene, representing the fold difference in mRNA expression in the treatment groups when compared with that in the vehicle control group.

### 4.3. Western Blotting

HepG2 cell lysates were isolated using ice-cold ProEX™ CETi protein extract solution (TransLab Biosciences, Daejeon, Republic of Korea) containing a protease and phosphatase inhibitor cocktail. ACC, p-ACC, AMPK, p-AMPK, β-actin, BiP, CHOP, FAS, GAPDH, IRE1α, and SCD1 antibodies were purchased from Cell Signaling Technology (Cell Signaling Technology, Inc., Danvers, MA, USA). Antibodies against ATF6, CD36, and PLIN2 were purchased from Abcam (Boston, MA, USA). The 4-HNE and PPARα antibodies were purchased from Abcam (Cambridge, MA, USA). Antibodies against ERK1/2, p-ERK1/2, JNK, p-JNK, and SREBP-1 were purchased from Santa Cruz Biotechnology, Inc. (Dallas, TX, USA). After primary antibody incubation, membranes were washed with TBS-Tween 20 (TBST; TransLab Biosciences) and incubated with horseradish peroxidase-conjugated secondary antibodies for 1 h at room temperature. Signals were visualized using an enhanced EZ-Western Lumi Pico detection kit (Dogen, Seoul, Republic of Korea) and the Azure c300 western blot imaging system (Azure Biosystems, Dublin, CA, USA).

### 4.4. Oil-Red-O Staining

To measure FFA-induced lipid accumulation in HepG2 cells, the Oil-Red-O staining solution was purchased from Sigma-Aldrich, Inc. (#O1391). Cells were seeded into a 6-well plate and pretreated with isoquercitrin for 6 h. The cells were treated with FFA containing oleic acid and palmitic acid (2:1) at a final concentration of 0.5 mM and cultured for 16 h. Lipid accumulation was determined using a previously reported Oil-Red-O staining method. The cells were thrice washed with phosphate-buffered saline (PBS) and fixed with 4% paraformaldehyde for 30 min. After fixation, the cells were thrice washed with PBS and stained with Oil-Red-O solution for 60 min at room temperature. The cells were washed again with PBS to remove any unbound stain. To quantify the Oil-Red-O content, dimethyl sulfoxide (DMSO) was added to each sample. After 5 min, the sample density was measured at 510 nm using a MULTISKAN GO reader (Thermo Fisher Scientific, Waltham, MA, USA) [83]. The difference in optical density was measured by normalization using total protein. 

### 4.5. Statistical Analysis

To establish statistical significance, collected data were analyzed by two-way ANOVA using GraphPad Prism version 5.0 software (GraphPad Software, San Diego, CA, USA). A *p*-value of ˂0.05 was deemed statistically significant.

## Figures and Tables

**Table 1:** List of human primers used for real-time RT-PCR.

Symbol	Primer Sequence (5′-3′)	
	Forward	Reverse
*GRP78*	TCGGCCGCACGTGGAATGAC	GCAGCTGCCGTAGGCTCGTT
*CHOP*	CAGAACCAGCAGAGGTCACA	AGCTGTGCCACTTTCCTTTC
*SCD1*	AGTTCTACACCTGGCTTGG	GTTGGCAATGATCAGAAAGAGC
*PPAR* *α*	CAATGCACTGGAACTGGATGA	GTTGCTCTGCAGGTGGAGTCT
*CPT1*	TCCAACTCACATTCAGGCAG	TTAAACATCCGCTCCCACTG
*FATP5*	TGATGGGACTTGTCGTTG	CCAGAAGCAGGAAGTAGAGAA
*GAPDH*	GGCGTCTTCACCACCATGGA	GCCTGCTTCACCACCTTCTT

## Data Availability

The data presented in this study are available in the article.
